# Supplementary material for: Content-rich biological network constructed by mining PubMed abstracts
Source: BMC Bioinformatics. 2004 Oct 8;5:147. doi: 10.1186/1471-2105-5-147 (PMC528731; doi:10.1186/1471-2105-5-147)
Supplement: Additional File 2 — The original results of the above study (non-essential files are deleted to keep the file size under the limit set by BMC bioinformatics). [file 1471-2105-5-147-S2.bz2 › chilibotAdditionalFile2/dip20/746ID7744737E18126/html/TOLA_TOLQ.html]

 


 **TOLA** and **TOLQ** 
  
Found 36 abstracts in PubMed, retrieved 20.  
 

 What does Google say? 
 PDF only 
| .edu only 

---

**Interactive relationship** (e.g. stimulation, inhibition, etc)

**Neutral relationship**- Role of TolR N terminal, central, and C terminal domains in dimerization and interaction with  **TolA**  and  **tolQ** .  Ref: 10419942 J Bacteriol, 1999
- The  **TolQ**  TolR proteins energize  **TolA**  and share homologies with the flagellar motor proteins MotA MotB.  Ref: 11722743 Mol Microbiol, 2001
- Cross linking experiments have shown that the  **TolA**  transmembrane domain interacts with  **TolQ**  and TolR.  Ref: 10419942 J Bacteriol, 1999
- These results confirmed the essential role of the transmembrane domain of  **TolA**  in triggering interactions with  **TolQ**  and TolR.  Ref: 9851983 J Bacteriol, 1998
- A point mutation within the third transmembrane TM segment of  **TolQ**  was found to affect the  **TolA**  Pal interaction strongly.  Ref: 11722743 Mol Microbiol, 2001
- The N terminal transmembrane domain of  **TolA**  anchors the protein to the cytoplasmic membrane and interacts with  **TolQ**  and TolR.  Ref: 9851983 J Bacteriol, 1998
- The TolR transmembrane domain was shown to be involved in the  **TolA**  TolR and  **TolQ**  TolR interactions, while TolR central and C terminal domains appeared to be involved in TolR dimerization.  Ref: 10419942 J Bacteriol, 1999
- Energy dependent conformational change in the  **TolA**  protein of Escherichia coli involves its N terminal domain,  **TolQ** , and TolR.  Ref: 11418549 J Bacteriol, 2001
- Therefore, the reduction in O7 LPS was attributed to the lack of a functional  **tolA**  gene, caused either by a direct mutation of this gene or by a polar effect on  **tolA**  gene expression exerted by the  **tolQ**  mutation.  Ref: 11069653 Mol Microbiol, 2000

**Non-interactive relationship** (e.g. studied together, co-existance, homology, etc.)

- The O7 deficient phenotype in the  **tolQ**  and  **tolA**  mutants was complemented with a plasmid encoding the tolQRA operon, but not with a similar plasmid containing a frameshift mutation inactivating  **tolA** .  Ref: 11069653 Mol Microbiol, 2000
- **TolQ** , TolR, and  **TolA**  are located in the cytoplasmic membrane where they interact with each other via their transmembrane domains.  Ref: 10633120 J Bacteriol, 2000
- However, strains with mutations in both tcpA and either  **tolQ** , tolR, or  **tolA**  were completely resistant to CTXphi infection.  Ref: 10692381 J Bacteriol, 2000
- The  **tolA**  and  **tolQ**  mutants showed reduced O7 LPS expression compared with the respective isogenic parent strains.  Ref: 11069653 Mol Microbiol, 2000
- This cluster was found to consist of seven genes in the order orf1  **tolQ**  tolR  **tolA**  tolB oprL orf2.  Ref: 10735848 J Bacteriol, 2000
- Suppressor mutant analyses have localized the  **TolQ**   **TolA**  interaction to the first transmembrane domain of  **TolQ**  and have shown that the third transmembrane domain of  **TolQ**  interacts with the transmembrane domain of TolR.  Ref: 10419942 J Bacteriol, 1999
- Mutations affecting the function of  **TolA**  resulted in a lack or an alteration in  **TolA**   **TolQ**  or TolR  **TolA**  interactions but did not affect the formation of  **TolQ**  TolR complexes.  Ref: 9851983 J Bacteriol, 1998
- Mutations in  **tolQ** , tolR,  **tolA** , tolB, or pal genes result in sensitivity to bile salts and the leakage of periplasmic proteins.  Ref: 10633120 J Bacteriol, 2000
- In this study, we show that both  **TolQ**  and TolR proteins are essential for the  **TolA**  Pal interaction.  Ref: 11722743 Mol Microbiol, 2001
- In the present study we cloned and mutagenized the orf1,  **tolQ** , tolR,  **tolA** , and tolB genes from P. putida KT2440, which were located upstream of the oprL gene.  Ref: 10940016 J Bacteriol, 2000
- Transmembrane domains of  **TolQ** , TolR and  **TolA**  interact in the cytoplasmic membrane, while TolB and Pal form a complex near the outer membrane.  Ref: 9701827 Mol Microbiol, 1998
- It is organized into two complexes, one near the outer membrane between TolB and PAL and one in the cytoplasmic membrane between  **TolA** ,  **TolQ** , and TolR.  Ref: 10419942 J Bacteriol, 1999
- Uptake of the mutated colicin U DTMVV depended on TonB and ExbB and still remained  **TolA**  and  **TolQ**  dependent, but no longer required TolB.  Ref: 9682486 FEMS Microbiol Lett, 1998
- **TolA** ,  **TolQ** , and TolR are inner membrane proteins, TolB is a periplasmic protein, and Pal, the peptidoglycan associated lipoprotein, is anchored to the outer membrane.  Ref: 11790745 J Bacteriol, 2002
- This change requires  **TolQ** , TolR, and a functional  **TolA**  N terminal domain.  Ref: 11418549 J Bacteriol, 2001
- Analysis of polar mutations, reverse transcriptase PCR assays, and transcriptional fusion constructs with a promoterless lacZ gene revealed that the genes are arranged in two operons orf1  **tolQ**  tolR  **tolA**  tolB and oprL orf2.  Ref: 12486055 J Bacteriol, 2003
- Bacteria carrying  **tolA** ,  **tolQ**  and tolR mutations kept their colicin insensitivity as spheroplasts, just as the resistant ones.  Ref: 12094727 Folia Microbiol Praha , 2002
- The Tol Pal system of the Escherichia coli envelope is formed from the inner membrane  **TolQ** , TolR and  **TolA**  proteins, the periplasmic TolB protein and the outer membrane Pal lipoprotein.  Ref: 11115123 Mol Microbiol, 2000
- The interactions of TolR with  **TolA**  and  **TolQ**  were confirmed, TolR was shown to dimerize, and the resulting dimer was shown to interact with  **TolQ** .  Ref: 10419942 J Bacteriol, 1999
- It was shown that the  **TolA** ,  **TolQ** , and TolR proteins play a critical role in the translocation of group A colicins and filamentous bacteriophage minor coat proteins g3p.  Ref: 11851406 Biochemistry, 2002
- Polar and nonpolar mutations of the P. putida  **tolQ** , tolR,  **tolA** , and tolB genes were generated in vitro by using the omega Km r interposon, which carries two transcriptional stop signals, or a promoterless xylE cassette, lacking any transcriptional stop signal, respectively.  Ref: 10940016 J Bacteriol, 2000
- In contrast, a polar  **tolQ**  mutation affecting  **tolA**  expression in VW187 caused slow growth rate and serum sensitivity in addition to reduced O7 LPS production.  Ref: 11069653 Mol Microbiol, 2000
- The Tol Pal system of Escherichia coli is composed of the YbgC,  **TolQ** ,  **TolA** , TolR, TolB, Pal and YbgF proteins.  Ref: 11994151 Mol Microbiol, 2002
- In Pseudomonas putida, the seven genes, orf1,  **tolQ** , tolR,  **tolA** , tolB, oprL, and orf2, which encode the proteins of this complex, are clustered in a 5.8 kb region of chromosomal DNA.  Ref: 12486055 J Bacteriol, 2003
- The  **TolQ**  TolR  **TolA**  proteins form a complex in the cytoplasmic membrane, while TolB and Pal interact near the outer membrane.  Ref: 10474183 FEMS Microbiol Lett, 1999
- Mutational analysis of the Escherichia coli K 12  **TolA**  N terminal region and characterization of its  **TolQ**  interacting domain by genetic suppression.  Ref: 9851983 J Bacteriol, 1998
- Transmembrane domains of  **TolQ** , TolR, and  **TolA**  interact in the cytoplasmic membrane, while TolB and Pal form a complex near the outer membrane.  Ref: 9851983 J Bacteriol, 1998
- A promoterless lacZ gene was introduced downstream of plasmid encoded  **tolQ**  and  **tolA** , and expression was monitored by measuring beta galactosidase activity of cultures.  Ref: 9603869 J Bacteriol, 1998
- V. cholerae strains with insertion mutations in  **tolQ** , tolR, or  **tolA**  were reduced in their efficiency of CTXphi uptake by 4 orders of magnitude.  Ref: 10692381 J Bacteriol, 2000
- DNA transfer requires the chromosomally encoded  **TolA** ,  **TolQ** , and TolR cytoplasmic membrane proteins.  Ref: 9537368 J Bacteriol, 1998
- **TolQ** , TolR, and  **TolA**  inner membrane proteins of Escherichia coli are involved in maintaining the stability of the outer membrane.  Ref: 11418549 J Bacteriol, 2001
- The predicted amino acid sequences of V. cholerae  **TolQ** , TolR,  **TolA** , and TolB showed significant similarity to the corresponding E. coli sequences.  Ref: 10692381 J Bacteriol, 2000
- This fragment was also detected in Delta  **tolQ** , Delta tolR, and  **tolA**  H22P mutants but, in contrast to the parental strain, was also seen in the absence of uncouplers.  Ref: 11418549 J Bacteriol, 2001
